# Application of the Kvaal method to CBCT reconstructed panoramic images for age estimation

**DOI:** 10.1007/s12024-024-00783-x

**Published:** 2024-01-25

**Authors:** Hatice Çelik, Mehmet Ali Kılıçarslan, Hatice Boyacioglu, Burak Bilecen

**Affiliations:** 1 Forensic Sciences Institute, Ankara University, Ankara, Turkey; 2 Graduate School of Health Sciences, Ankara University, Ankara, Turkey; 3 Department of Prosthodontics, Faculty of Dentistry, University of Ankara, Ankara, Turkey; 4 Department of Dentomaxillofacial Radiology, Faculty of Dentistry, Hacettepe University, Ankara, Turkey; 5 Department of Anatomy, Faculty of Medicine, Ankara Medipol University, Ankara, Turkey

**Keywords:** Age estimation, Cone-beam computed tomography, Forensic anthropology, Kvaal method, Teeth

## Abstract

As the teeth are more durable than other parts of the skeleton, they provide valuable data for age estimation. Age estimation from adult teeth is mainly based on secondary dentin production. The present study aimed to devise a regression formula for age estimation specific to the Anatolian population using the Kvaal method on CBCT reconstructed panoramic images. In total, 201 individuals aged between 20 and 69 were divided into two groups: data from the study group (*n* = 101) were used to create the regression formulae, and data from the control group (*n* = 100) were used to test the formulae. Pearson’s correlation coefficients and linear regression analyses were performed. Maxillary teeth provided more accurate age estimates than mandibular teeth. The regression formulae derived in this study are found to be statistically applicable and reasonably accurate. However, these results should be interpreted with caution.

## Introduction

Chronological age is that from the date of birth. Low error and accurate estimation of the chronological age are of the utmost importance for correct identification. Time-dependent disruptions of soft and hard tissues are commonly analyzed for age estimation. However, differences between the chronological and estimated age occur due to individual, environmental, and genetic factors [1]. Age estimation can be carried out using a variety of methods, such as analysis of ossification centers in the long bones of the extremities and radiographic evaluation of hand–wrist bones [2], abrasions of the joint surface [3], closure of the symphysis pubis and sutures of cranial bones [4], and the surface between the ribs and sternum [5].

In events such as fires, explosions, and terror attacks, many parts of the skeleton may be damaged; identification can be difficult because of the vulnerability of bone to high temperatures. Teeth are more resistant to high temperatures than bone tissue and may, therefore, be the only material available for identification in some cases. Thus, teeth are important for age estimation, as evidenced by the numerous studies using teeth for this purpose. Gustafson [6] developed an age estimation method based on histological changes in adult teeth. The pulp space decreases due to secondary dentin production, regardless of gender [7, 8]. Cameriere et al. [9] determined the ratio between the pulp area of the maxillary canines and total tooth area; they also analyzed the correlation between this ratio and age. In mandibular premolars and molars, the ratio between the pulp chamber height and crown height has been investigated; no statistically significant difference was reported according to gender [10, 11].

Kvaal et al. [12] developed a regression formula for age estimation based on secondary dentin formation measurements using dental periapical radiographs. The measurements performed included maximum tooth length, maximum pulp length, maximum root length, root and pulp width at the enamel–cementum junction (A), root and pulp width at the mid-root level (C), and the root and pulp width halfway between levels A and C. Based on these measurements, tooth/root length, pulp/root length, and pulp/tooth length ratios were calculated, as well as the pulp/root width ratio at three levels. Regression formulae evaluated the correlations between these ratios and age; the teeth measurements showing the strongest correlations with age were those of the maxillary central incisor, lateral incisor, second premolar, lateral incisor, canine, and first premolar in the mandible. There were no differences between right or left teeth; thus, teeth on either side could be used for measurement [12]. Studies investigated the applicability of this method on different populations with different imaging techniques, such as panoramic images (13–14).

The present study aimed to develop an age estimation formula specific to the Anatolian population based on secondary dentin measurements of the Kvaal method using CBCT reconstructed panoramic images. The null hypothesis is that analysis of cone-beam computed tomography (CBCT) images can facilitate age estimation based on the Kvaal method.

## Materials and method

This study was conducted with the approval of the Non-Interventional Clinical Research Ethics Board of Hacettepe University (approval date: 25.02.2020; approval number: 16 969 557–382).

Archives of CBCT scans acquired using the I-CAT system (Imaging Sciences International, Hatfield, PA, USA) were analyzed. Images of the maxilla and mandible (voxel size = 0.200) were evaluated. CBCT images of normal adults (aged > 20 years) meeting the eligibility criteria were included in the study. All participants were required to have sound teeth. Images were examined using the bundled I-CAT Vision software (version 1.9.3.14; Imaging Sciences International). All measurements were made on reconstructed panoramic images of CBCT scans.

In this study, 208 individuals were included in the initial sample. Seven individuals were excluded from the study because the pulp width was too narrow for accurate CBCT measurements. The final sample of 201 individuals was divided into study and control groups. The study group included 101 individuals and was used to create the regression formulae, and the control group consisting of 100 individuals was used for testing the regression formulae.

Teeth were selected according to the method defined by Kvaal et al. [12]. Maxillary central incisors, lateral incisors, second premolars, mandibular lateral incisors, canines, and first premolars were selected because they tend to show the strongest correlations with age. Teeth that were fully visible and in functional occlusion were included. The exclusion criteria were as follows: presence of caries, restoration, root canal treatment, developmental anomaly, and tooth pathology.

**Fig. 1:**
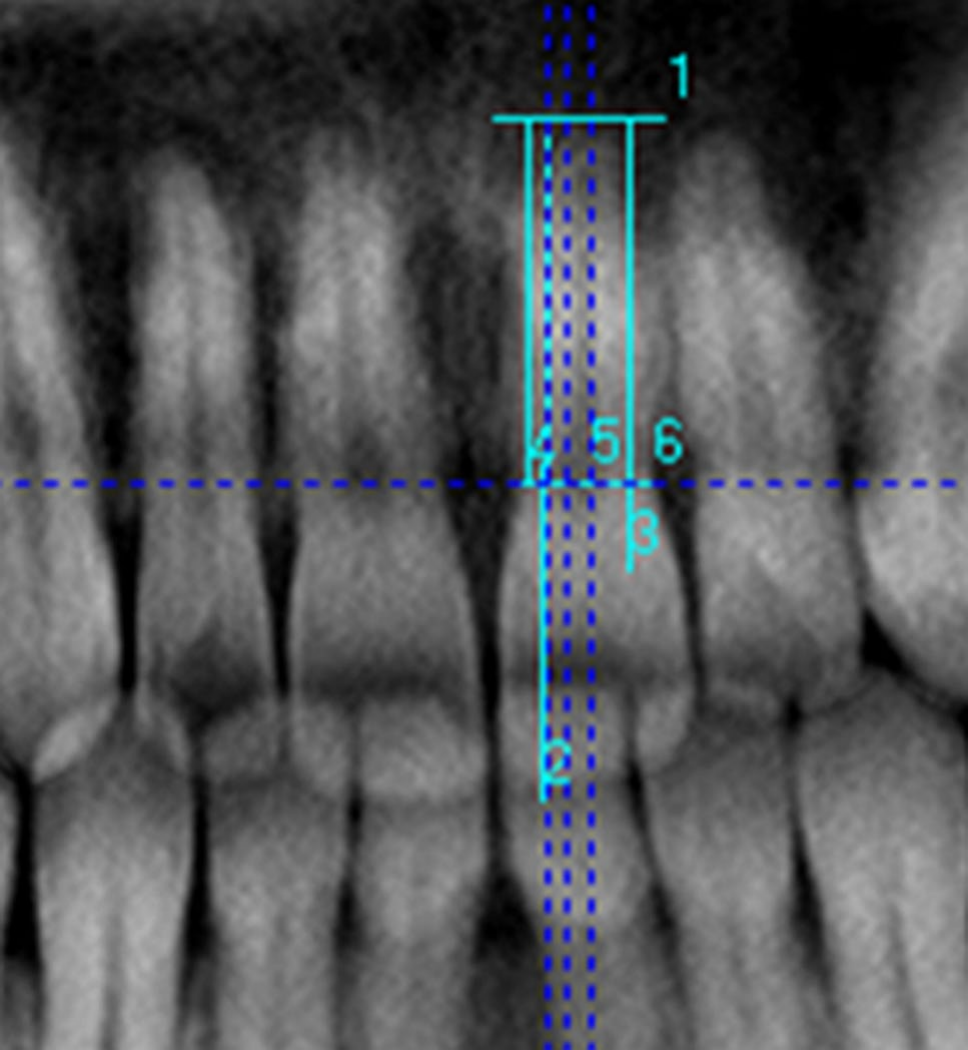
Length measurements of the teeth

The observer manually drew dental arch lines to generate panoramic reconstructed images of the maxilla and mandible. Tooth selection was based on reconstructed panoramic images of CBCT scans. If present, teeth on the left side of the jaw were analyzed; in their absence, their right-sided counterparts were analyzed. The location of the apex, the longest length of the tooth, and the highest point of the chamber were determined on cross-sectional images; these points were used as a reference for the measurements made on the CBCT reconstructed panoramic images. Due to the inclination of the axes of the teeth toward the mesiodistal direction, the length measurements were made parallel to the pulp canal to approximate the actual length as closely as possible. Because of the labiolingual position of the teeth, the point at which the enamel–cementum line was closest to the apex on the lingual side on cross-sectional images was used as a reference to determine the location of the cervical line (Fig. 1).

**Fig. 2:**
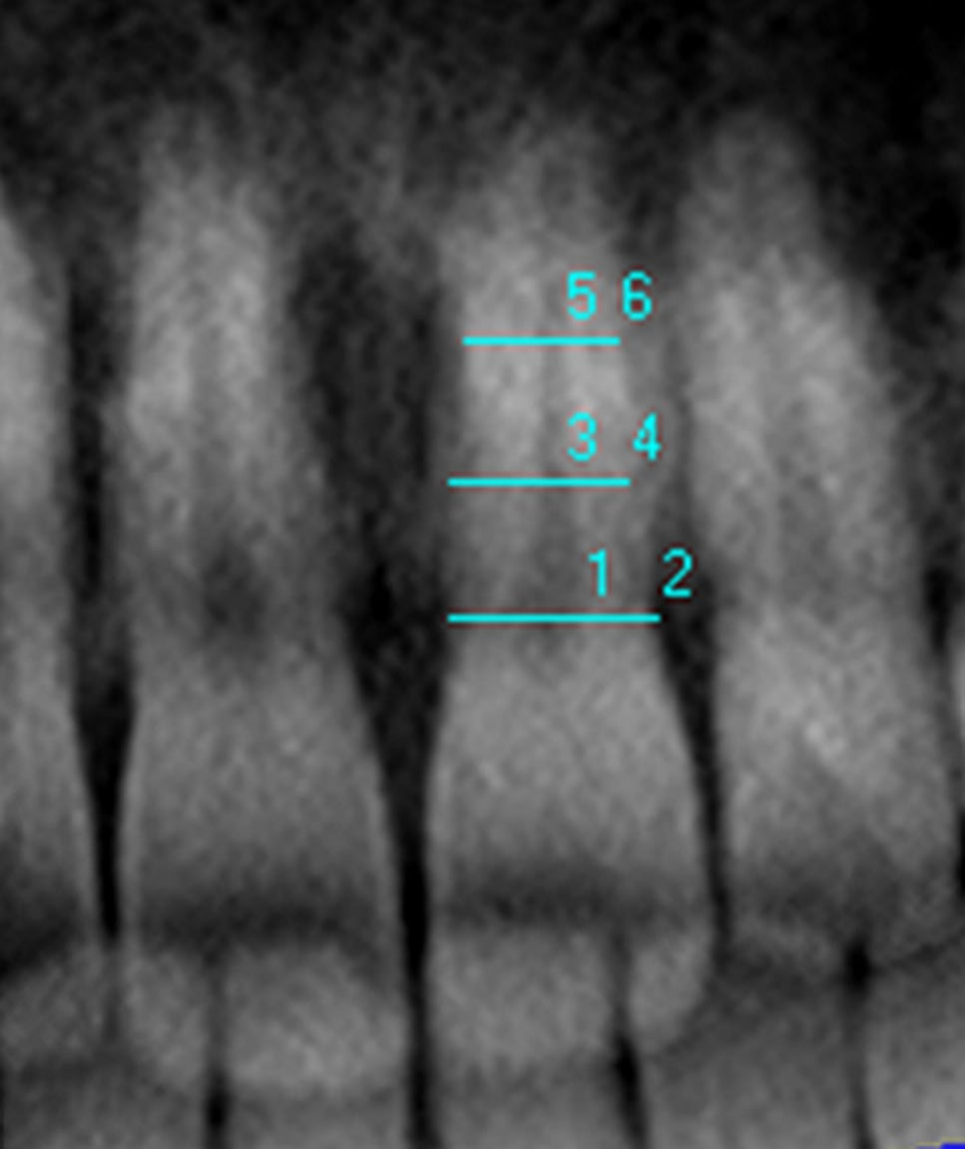
Width measurements of the teeth

The width ratios were as follows: *A*, ratio between the pulp and root widths at the enamel–cementum junction level; *C*, ratio between the pulp and root widths at the midpoint between the enamel–cement junction and root apex; and *B*, ratio between the pulp and root widths at the midpoint of *A* and *C*. The length ratios were as follows: *T*, ratio between the tooth and root lengths; *P*, ratio between the pulp and root lengths; and *R*, ratio between the pulp and tooth lengths. *W* represents the average width ratio at the *B* and *C* levels. *L* is the average value of the *P* and *R* length ratios (Fig. 2).

Measurements were carried out by two researchers with 10 and 24 years of experience. To determine the intra-observer consistency, 15 days after completion of the measurements, measurements were remade in 40 randomly selected individuals from each age group (20% of all individuals measured); inter-observer consistency was evaluated based on measurements of these individuals made by a second researcher.

Statistical analysis was conducted using SPSS software (version 21.0; IBM Corp., Armonk, NY, USA). The paired *t*-test was performed to assess intra- and inter-observer agreement. Pearson’s correlation coefficients were calculated to evaluate the relationships between age and the various ratios. Linear regression analyses were performed with age, the ratios, and gender as independent variables. A stepwise procedure was applied in these analyses, and only independent variables meeting the *p* < 0.05 significance criterion were included. A *t*-test of conjugated samples was used to determine whether the estimated and chronological mean age differed significantly in the control group.

## Results

The participants ranged in age from 20 to 69 years (mean age, 38.58 ± 12.64 years); there were 111 (55.2%) females and 90 (44.8) males. The age and gender data are presented in Table 1. There was no difference between intra- and inter-observer agreement (*p* > 0.05).

**Table 1:** Age and gender distribution of the participants

Age groups	Female	Male	Total
20–29	34	31	65
30–39	30	16	46
40–49	25	18	43
50–59	18	17	35
60–69	4	9	12
Total	111	90	201

The Pearson correlation coefficients are shown in Table 2. The *P* and *A* parameters showed the strongest correlations with chronological age. The *P* ratio had the strongest correlation with chronological age for the maxillary central incisors (*r* = 0.56), and the *A* ratio had the strongest correlation with chronological age for the maxillary central (*r* = 0.38) and maxillary lateral teeth (*r* = 0.36).

**Table 2:** The correlation coefficients between the ratios of the measurements obtained from the study group and age (*n* = 101) (*p < 0.05, **p < 0.01)

Tooth	11/21	12/22	15/25	42/32	43/33	44/34	Maxilla	Mandibula	Both jaws
Tooth	−0.09	−0.03	0.04	−0.04	0.10	−0.07	−0.04	−0.01	−0.02
Pulp	−0.29**	−0.25*	−0.03	−0.19	0.01	−0.11	−0.24*	−0.11	−0.19
Root	0.08	0.11	0.20*	0.14	0.24*	0.09	0.17	0.19	0.19
A_Pulp	−0.67**	−0.63**	−0.53**	−0.58**	−0.45**	−0.55**	−0.71**	−0.62**	−0.72**
A_Root	−0.03	0.05	0.04	−0.07	−0.01	−0.01	0.03	−0.03	< 0.001
B_Pulp	−0.60**	−0.51**	−0.12	−0.35**	−0.14	−0.48**	−0.43**	−0.35**	−0.49**
B_Root	0.03	0.12	0.05	0.10	−0.07	−0.08	0.09	−0.07	−0.03
C_Pulp	−0.52**	−0.40**	−0.06	−0.40**	−0.27**	−0.35**	−0.34**	−0.44**	−0.44**
C_Root	0.14	0.20*	0.03	−0.02	−0.11	−0.03	0.18	0.04	0.13
*P*	−0.56**	−0.40**	−0.44**	−0.43**	−0.39**	−0.35**	−0.56**	−0.45**	−0.58**
*T*	−0.28**	−0.17	−0.23*	−0.19	−0.32**	−0.29**	−0.35**	−0.31**	−0.40**
*R*	−0.39**	−0.36**	−0.12	−0.02	−0.14	−0.11	−0.37**	0.16	−0.29**
*A*	−0.66**	−0.64**	−0.55**	−0.59**	−0.46**	−0.52**	−0.72**	−0.60**	−0.71**
*B*	−0.60**	−0.56**	−0.13	−0.34**	−0.21*	−0.46**	−0.49**	−0.37**	−0.53**
*C*	−0.57**	−0.48**	−0.03	−0.32**	−0.37**	−0.36**	−0.33**	−0.48**	−0.46**

Multiple regression formulae were generated for each individual tooth, and for three mandibular teeth, three maxillary teeth, and all six teeth. Gender was not included in any regression formula, as it had a *p*-value > 0.05 (Table 3). The mean, standard deviation, minimum, and maximum values of the estimated residuals obtained in the regression analyses are presented in Table 4. The regression formulae with the highest coefficients of determination (*r*^2^) were those for all six teeth and three maxillary teeth. The formulae for the three maxillary teeth had higher predictive accuracy than those for the three mandibular teeth. The formulae for the maxillary central teeth had higher predictive accuracy than those for the other teeth. The regression formulae for mandibular and maxillary lateral teeth had similar coefficients of determination. The formulae for the maxillary premolar, mandibular canine, and mandibular premolar teeth also had similar coefficients, all of which were low.

**Table 3:** Derived regression models from the study group (*n* = 101)

	Equation	*r*^2^	SEE (years)
6 teeth in both jaws	Age = 109.16 – 25.47(T) – 153.43(*A*) – 46.79(*B*)	0.54	8.15
3 maxillary teeth	Age = 96.92 – 20.40(*T*) – 157.37(*A*)	0.54	8.18
3 mandibular teeth	Age = 72.35 – 147.47(*A*) – 79.28(*C*)	0.38	9.50
Teeth (independent)			
11/21	Age = 92.62 – 28.03(*P*) – 72.62(*A*) – 61.80(*C*)	0.51	8.40
12/22	Age = 60.85 – 127.62(*A*)	0.41	9.28
15/25	Age = 57.00 – 124.92(*A*)	0.29	10.15
42/32	Age = 85.00 – 22.17(*P*) – 108.28(*A*) – 26.23(*B*)	0.40	9.31
43/33	Age = 100.21 – 27.91(*T*) – 87.49(*A*) – 53.18(*C*)	0.26	10.35
44/34	Age = 64.49 – 92.35(*A*) – 77.22(*B*)	0.29	10.12

*r*^2^ coefficients of determination, *SEE* standard error of estimate

**Table 4:** Statistics on residuals obtained in regression formulae (*n* = 101)

	Mean	*SD*	min	max
6 teeth in both jaws	38.16	8.98	20.79	62.13
3 maxillary teeth	38.16	8.91	20.46	61.24
3 mandibular teeth	38.16	7.52	20.87	57.74
Teeth (independent)				
11/21	38.16	8.75	19.86	62.74
12/22	38.16	7.73	18.42	56.58
15/25	38.16	6.56	19.53	52.38
42/32	38.16	7.81	14.74	65.71
43/33	38.16	6.41	18.70	53.77
44/34	38.16	6.68	22.29	54.35

*SD* standard deviation

Measurements from the control group were utilized to assess the estimation accuracy of the regression formulae. The ratios obtained from the control group are presented in Table 5. The differences between the estimated and chronological mean ages were not statistically significant for any formulae (Table 6).

**Table 5:** Estimated mean age from regression models in the control group (*n* = 100)

	Estimated mean age (*SD*)	SEE (years)
6 teeth in both jaws	35.63 (15.67)	9.56
3 maxillary teeth	36.95 (9.27)	7.97
3 mandibular teeth	36.45 (10.81)	9.96
Teeth (independent)		
11/21	36.83 (15.57)	8.58
12/22	38.55 (8.10)	8.86
15/25	36.55 (6.53)	9.86
42/32	36.45 (17.75)	11.19
43/33	37.88 (7.95)	9.49
44/34	37.82 (5.99)	9.84

**Table 6:** Differences between the estimated mean age and the real mean age (*n* = 100). Real mean age = 38.80 (*SD* = 13.17)

	Estimated mean age (*SD*)	Difference (*SD*)	*t*	*p*
6 teeth in both jaws	35.63 (15.67)	3.07 (16.35)	1.88	0.06
3 maxillary teeth	36.95 (9.27)	1.90 (9.96)	1.90	0.06
3 mandibular teeth	36.45 (10.81)	2.35 (13.56)	1.73	0.09
Teeth (independent)				
11 > 21	36.83 (15.57)	2.10 (14.33)	1.46	0.15
12 > 22	38.55 (8.10)	0.42 (10.76)	0.39	0.70
15 > 25	36.55 (6.53)	2.25 (11.64)	1.92	0.06
42 > 32	36.45 (17.75)	2.35 (20.28)	1.15	0.25
43 > 33	37.88 (7.95)	0.91 (11.44)	0.80	0.43
44 > 34	37.82 (5.99)	0.98 (11.64)	0.84	0.41

## Discussion

For accurate age estimation, error must be minimized. Methods used for age estimation in adulthood are based on age-related structural changes, such as secondary dentin formation and abrasions on the tooth surface; thus, regression formulae that reflect these changes are required [10–12]. The size of the pulp chamber decreases with age due to secondary dentin formation. Kvaal et al. [12] measured changes in the pulp chamber and pulp canal due to secondary dentin formation. In this study, pulp height and pulp canal width ratios were calculated in relation to constant parameters such as root height and width, and the correlations of the ratios with chronological age were investigated. A regression formula that included the ratios showed that they were strongly correlated with chronological age. Population-specific regression formulae are necessary to reduce error in age estimations for populations different from that on which a given formula was based [13–17].

The present study aimed to create a regression formula for age estimation specifically for the Anatolian population, based on measurements performed on CBCT images using the Kvaal method. The null hypothesis, i.e., that making measurements on CBCT images is advantageous for age estimation, was supported. Archived images of the Department of Oral, Dental, and Maxillofacial Radiology, Faculty of Dentistry, Hacettepe University were used. Erbudak et al. evaluated conventional panoramic images from the same archive [17]. Similar to our study, they reported that maxillary central and mandibular lateral teeth measurements showed the strongest correlations with age. However, the regression formulae derived in their study showed lower accuracy than those obtained in the current one; their coefficients of determination ranged between 0.035 and 0.345 [17], whereas in the current study, they had values of 0.26–0.54. The stronger correlations in this study could be attributed to the imaging technique and image quality; CBCT imaging is advantageous for teeth measurements because it overcomes the problems associated with panoramic imaging, such as rotation, magnification, and superposition. Therefore, CBCT can provide clear and error-free high-resolution images. Additionally, the high image quality provided by CBCT helps observers clearly identify the anatomic landmarks of the tooth, which enhances measurement precision. These factors may explain the relatively strong correlations between age and the measured ratios obtained in this study.

Kvaal et al. [12] reported that the “W-L value” was correlated with age. However, in the current study, we used the predictors that showed the strongest correlations with chronological age to develop accurate and applicable regression formulae; the W-L value was not among those predictors. Unlike the study of Kvaal et al. [12], which included a population aged 20–87 years, our study included individuals aged 20–69 years. We used 69 years as the upper age limit because older individuals are more likely to undergo restoration, root canal therapy, and tooth extraction; thus, very few individuals aged ≥ 70 years would have met the study inclusion criteria. Notably, in their regression formulae based on the Kvaal method, Karkhanis et al. [14] reported that only the W-L values for the maxillary central and lateral teeth correlated with age; however, they did not include predictors with stronger correlations in their regression formula. Another study reported a high correlation coefficient for W-L, consistent with Kvaal et al. [18]. However, it is difficult to explain this result; it may be related to differences in tooth dimensions among populations, which would affect the ratio and correlation values.

Akay et al. [19] evaluated a population similar to that which provided the archived images analyzed in this study and compared the utility of pulp/tooth volume ratios and the Kvaal method for age estimation; while the W and L values both correlated with age, the correlation for the W-L value was not examined. Their regression formulae were based on the W-L value and Kvaal’s original formula. In the regression formula derived for six teeth, a high standard error of the estimate (SEE) value of 12.75 years was obtained, which is not suitable for age estimation. In our study, the SEE value for the regression formula developed for six teeth was 8.15 years. However, Akay et al. [19] reported a SEE value of 5.44 years for their regression formula of maxillary lateral teeth, which is satisfactory for forensic studies.

The first study to apply the Kvaal method to CBCT images was that of Penaloza (2016), in which measurements were performed on sagittal and coronal images in the buccolingual and mesiodistal directions [15]. The buccolingual measurements reportedly had stronger correlations with chronological age than the mesiodistal measurements. However, the use of buccolingual measurements did not result in an improvement in the results previously obtained for the same population using panoramic radiographs.

In the present study, the measurements were performed on CBCT reconstructed panoramic images rather than on multiplanar images; multiplanar images were used only to confirm the reference points in the reconstructed panoramic image. The derived regression formulae showed high accuracy but do not support the results of previous studies. The strong correlations observed herein could be attributed to the increased accuracy of measurements made on CBCT reconstructed panoramic images compared to conventional panoramic images.

Another study [20] applied the Kvaal method to central teeth appearing on sagittal CBCT images; the regression formulae derived from buccolingual measurements were not useful due to the high SEE values. Similarly, Pires et al. [21] performed the buccolingual and mesiodistal measurements proposed by Kvaal et al. [12] on central, lateral, and canine (maxillary and mandibular) teeth on CBCT images; the buccolingual measurements of the maxillary central teeth had the highest correlation coefficient with age. However, the derived regression formulae reported were not suitable for forensic studies [21]. Another study [22] applied the Kvaal method to cross-sectional CBCT images from an Iranian population; the derived formulae were reasonably accurate, but the original formula of Vossoughi et al. was not satisfactory [22].

Roh et al. [23] reported a high SEE value for the regression formula presented by Kvaal et al. [12]. In their correlation analysis, while length measurements did not correlate with age, width measurements showed strong correlations. These results are similar to those reported by Mittal et al. [24] and Patil et al. [25]; they also derived a high SEE value in their regression formula based on the Paewinsky method and sum of width ratios. In both studies, the most accurate chronological age estimations were obtained using regression formulae for three maxillary teeth and six teeth. Notably, these results [24, 25] are consistent with those obtained in the current study.

In the present study, the regression formulae were developed using the data of one group and tested using the data of another group. In other words, the validity of the regression formulae was tested. Similar to the current study, other researchers tested the validity of their regression formulae using a control group [13, 14].

In an Indian study, Kanchan-Talreja et al. [26] reported high SEE values for regression formulae developed based on parallel and angled measurements made on periapical radiographic images. Chandan et al. [18], Roh et al. [23], and Rajpal et al. [27] concluded that the Kvaal method had an acceptable margin of error. These studies show the importance of creating population-specific formulae; more studies are needed in countries with large and diverse ethnic groups.

Qu et al. [28] applied the Kvaal method in a subadult group aged between 15 and 21 and combined this method with the third molar mineralization method. The combined regression formula increased the coefficients of determination value from 0.05 to 0.51. These results suggest combining the Kvaal method with different dental age estimation methods may improve the accuracy of age estimation.

Kazmi et al. [29] reviewed the dental age estimation studies using the Kvaal method and compared the accuracy of the measurements of the Kvaal method. The results showed better correlation values for width measurements than length measurements. They suggested focusing on width measurements for creating formulae. Similarly, the width measurement of A showed the strongest correlation with age in the present study and was used to derive the regression formula. The result of the study mentioned above makes the age estimation with the Kvaal method simpler and faster, shortening measurement time. However, with a small sample study size (22 studies from 10 different populations), caution must be applied, as the findings might not be applicable to each population. Further studies, which take width measurements into account, will need to be undertaken.

Pereira de Sousa et al. [30] assessed the use of artificial intelligence in dental age estimation and compared the Kvaal method’s accuracy using linear regression analysis and machine learning algorithms. Machine learning algorithms presented lower mean error of the estimate values. These results implicate the potential use of artificial intelligence for improving the accuracy of age estimation.

In the present study, approximately 5000 CBCT scans were evaluated to identify individuals with the six teeth of interest who satisfied the inclusion criteria. Reviewing the CBCT scans was the most time-consuming part of the research process. Because the root pulp width could not be measured due to the pulp height being below the A point in individuals aged ≥ 70 years, these individuals were excluded from the study, which resulted in a small sample of older adults. Measurements were performed using cross-sectional and CBCT reconstructed panoramic images. The software used has a simple user interface that allows for easy manipulation by the observer. However, switching between two display screens during measurements is relatively time-consuming. Despite linear measurements made on CBCT reconstructed panoramic images being accurate and free of error, radiation exposure is a disadvantage of CBCT. In future studies, the applicability of other methods to CBCT images from the Anatolian population should be evaluated, and the method that yields the smallest amount of error should be determined.

## Conclusion

The present study investigated the relationship between chronological age and secondary dentin deposition on CBCT reconstructed panoramic images in the Anatolian population using the Kvaal method. Maxillary teeth provided more accurate age estimates than mandibular teeth. Although the results obtained using the regression formula derived in our study indicated that the Kvaal method is reasonably accurate for age estimation of the Anatolian population, these results should be interpreted with caution. CBCT images allow for precise measurement of secondary dentin deposition, providing clear images of three-dimensional tooth anatomy on different planes.

## Key points

1. Kvaal et al. developed a regression formula for age estimation based on secondary dentin formation measurements.
2. Based on these measurements, the present study aimed to develop an age estimation formula on CBCT images specific to the Anatolian population.
3. The regression formulae derived in this study are found to be reasonably accurate. Maxillary teeth provided more accurate age estimates than mandibular teeth.
4. CBCT images allow for precise measurement of secondary dentin deposition, providing clear images of three-dimensional tooth anatomy on different planes.

## Data Availability

Data for this study can be requested by contacting the corresponding author.
